# State anxiety, uncertainty in illness, and needs of family members of critically ill patients and their experiences with family-centered multidisciplinary rounds: A mixed model study

**DOI:** 10.1371/journal.pone.0234296

**Published:** 2020-06-09

**Authors:** Jiyeon Kang, Young-Jae Cho, Seunghye Choi

**Affiliations:** 1 Department of Anthropology, University of Virginia, Charlottesville, VA, United States of America; 2 Department of Internal Medicine, Division of Pulmonary and Critical Care Medicine, Seoul National University Bundang Hospital, Bundang-gu, Seongnam, Korea; 3 College of Nursing, Gachon University, Yeonsu-gu, Incheon, Korea; Nazareth Hospital, UNITED STATES

## Abstract

This study aimed to determine whether family-centered multidisciplinary rounds could alleviate anxiety and uncertainty in illness and meet needs for critically ill patients’ families. A family-centered multidisciplinary rounds protocol was developed identifying needs of critically ill patients’ families, and family experiences were reviewed through in-depth interviews. A sequential mixed-methods study was utilized, combining survey data and semi-structured interviews in a tertiary medical intensive care unit in South Korea. A structured questionnaire assessed needs, anxiety, and uncertainty in illness for 50 participants. Interview data of 10 participants were analyzed using grounded theory. Assurance was the highest family need, followed by information need. Family needs differed according to gender, relationship to the patient, and length of intensive care unit stay. Participants reported family-centered multidisciplinary rounds provided a sense of relief, a chance to listen to medical staff, and a chance to provide medical staff with comprehensive information about patient care. Proximity needs were found to have a positive correlation with state anxiety, while comfort needs had a negative correlation with uncertainty in illness. Families reported family-centered multidisciplinary rounds were positive, useful experiences. Thus, standardization of family-centered multidisciplinary rounds is needed to meet families’ various needs.

## Introduction

Intensive care unit (ICU) admissions are increasing at a fast pace, as indications for more available life support systems continue to expand, due to the aging population and recent advances in medical technology. South Korea had a total of 10,127 ICU beds in 2016, and more than 300,000 patients are admitted annually to ICUs for intensive treatment [1]. Although a growing number of patients seek active involvement in making decisions about their own healthcare [2], critically ill patients in ICUs are likely to be incapable of proactively participating in discussions regarding treatment plan and decision-making due to various medical conditions. Consequently, family members of patients in ICUs play a crucial part in substitute decision-making, psychological support, and follow-up care [3, 4]. Despite their significant role, families of critically ill patients also experience structural, emotional, and financial crises when their relatives are admitted to the ICU [5]. Particularly, as critically ill patients are prone to a wide variety of acute and life-threatening complications, their families feel anxiety caused by uncertainty of disease progression [6]. Thus, it is necessary to pay attention to family members of critically ill patients to ensure effective implementation of family-centered care [7].

Families of critically ill patients have a broad range of needs. It has been reported that family members showed enhanced decision-making capacity and less post-traumatic stress disorder symptoms when their needs were satisfied [8]. Also, it has been reported that family members of critical care patients placed great importance on needs for assurance, proximity, and information [9].

Facilitating communication between families of critically ill patients and medical staff can help meet the family’s needs, while also serving as an important factor in medical decision-making for critically ill patients. In the last decade, family-centered multidisciplinary rounds have been developed as new measures to stimulate the families’ engagement in conversations with the medical practitioners and to improve the quality of communication, patient satisfaction, post-discharge planning, and patient safety [10, 11, 12]. However, in Korea, only very few studies have explored this approach [13], and a protocol for family-centered multidisciplinary rounds has not yet been established.

Therefore, this study aimed to: (1) develop a family-centered rounds protocol that engages families members of critically ill patients in multidisciplinary rounds; (2) conduct a survey to identify their needs, state anxiety, uncertainty in illness of patients; and (3) and conduct semi-structured interviews with family members to better understand their experiences. The ultimate goal of this study was to determine if family-centered multidisciplinary rounds could contribute to alleviating state anxiety and uncertainty in families of critically ill patients, while also satisfying their needs.

## Methods

### Research design

This study conducted a mixed-model analysis by combining survey data and semi-structured interviews to identify variables related to critical care family needs, anxiety, and uncertainty in illness. All participants responded to questionnaires regarding critical care family needs, anxiety, and uncertainty in illness, and some of them were interviewed after they attended family-centered multidisciplinary rounds.

### Participants and data collection

A total of 50 family members of critically ill patients participated in this study between October, 2018 and May, 2019. The research site was a medical intensive care unit (MICU) in a tertiary hospital in South Korea. Inclusion criteria for family members were as follows: (1) those whose relative was in the ICU for ≥ 48 hours, (2) aged ≥ 18 years, and (3) those who visited the patient ≥ once per week. Of the 87 family members who agreed to participate, 50 were included in the final analysis. Those whose relative died during the research period (n = 12) and those who submitted incomplete questionnaires (n = 25) were excluded. 10 family members also agreed to participate in family-centered multidisciplinary rounds and semi-structured interviews.

A recruitment flyer was posted on a bulletin board at the ICU entrance during the study period. Additionally, nurses and residents directly contacted potential participants to ask if they would be willing to take part in the study. Those who voluntarily expressed their willingness to participate were recruited. Participants were asked to complete questionnaires. The 10 participants who agreed to attend family-centered multidisciplinary rounds were interviewed by a member of this research team after rounds ended. Interviews began with an open-ended question: “Can you tell me about the day the patient was admitted to the ICU?” The interviews included questions on the following: (1) psychological, physical, and financial distress faced by family members during ICU admission; (2) ways of supporting and providing care to patients; (3) evaluation of ICU staff and quality of care; and (4) evaluation of family-centered multidisciplinary rounds.

### Instruments

Information was collected regarding participants’ and patients’ genders and ages, family members’ relationship to the patient, cohabitation with the patient, length of stay (LOS), cause of admission, and Acute Physiology, Age, Chronic Health Evaluation II (APACHE II) scores [14]. In the case of interview participants, information regarding educational level and religion was also collected.

Family needs were assessed using the Korean version of the Critical Care Family Needs Inventory (K-CCFNI), originally developed by Molter [15] and Leske [16]. The K-CCFNI consists of 45 items: assurance (7), information (9), proximity (9), comfort (6), and support (14). In a previous study, goodness-of-fit indices for each subscale all exceeded their recommended acceptance levels [9]. In this study, mean scores for each subscale and mean total scores were calculated and compared.

State anxiety was assessed using the State Anxiety Inventory-X-1 as a standardized instrument for measuring anxiety. This inventory was originally developed by Spielberger [17], and then later translated into Korean [18]. All items are rated on a 4‐point Likert scale, and the item scores are summed to produce a total score ranging from 20 to 80. A 20~51 score indicates a state without anxiety, and a score of above 51 means that with state anxiety. To assess uncertainty in illness, we used the Mishel Uncertainty in Illness Scale (MUIS), which was originally developed by Mishel [19], and modified for use in a Korean population by Park [20] and Kim [21]. In terms of family needs, state anxiety, and uncertainty of illness, Cronbach’s alphas of the three scales were calculated.

### Family-centered multidisciplinary rounds protocol

The multidisciplinary team consisted of two physicians (attending physician, resident physician), two nurses (attending nurse, nurse practitioner), a pharmacist, and a social worker. Family members participated in rounds with the team. Based on previous studies, the family-centered multidisciplinary protocol was designed as follows: 1) obtaining a signed consent form prior to family-centered multidisciplinary rounds; 2) a social worker’s consultation with family members regarding their financial status and any available support programs to which they could apply; 3) introducing team members to family members at the commencement of rounds; 4) reporting by an attending nurse, a resident, a pharmacist, and a social worker in sequence, the patient’s current clinical condition, future plans for treatment, rationale for clinical judgment of treatment effects, goals of nursing care, and family preferences [22, 23]; and 5) providing family members with a question and answer session at the end of rounds [24].

### Data analysis

Survey data were analyzed using IBMⓇ SPSS version 23.0 (IBM Corp, Armonk, NY, USA). Key variables such as total score and subscale scores of needs, state anxiety, and uncertainty were tested for normality using Kolmogorov-Smirnov Z values. General characteristics and needs of critically ill patients and their families, state anxiety, and uncertainty in illness were presented in terms of real numbers, percentages, means, and standard deviations.

Needs of families of critically ill patients, state anxiety, and uncertainty in illness according to the families’ general characteristics were compared using Mann-Whitney U tests and t-tests. Correlations between ICU LOS, APACHE II scores and critical care family needs, state anxiety, and uncertainty in illness were obtained using Pearson’s correlation coefficients.

Semi-structured interview data were analyzed based on grounded theory [25]. Two investigators independently read and re-read the transcripts and field notes and generated codes and themes, which were combined into similar codes after interviews were completed. Each code was accompanied by a memo on the background and details of the code. Once the process of coding was complete, the two investigators examined each other’s coding results and modified code and theme structures through on-going discussions. After data collection was complete, all investigators re-examined the results from the first round of coding. Finally, the final codes and themes were created.

This study referred to the interview research checklist proposed by Tong, Sainsbury, and Craig [26], to ensure the reliability and validity of the qualitative research. To capture participants’ responses as much as possible, non-verbal communication, such as hesitation in responding and gestures exhibited during interviews, were recorded and transcribed. The investigator in charge of conducting interviews attended the family-centered multidisciplinary rounds so as to better understand the broader context in which participants were positioned. This research team consisted of researchers from a wide range of professions—an ICU attending physician, professor of nursing with critical care experience, and doctoral candidate in medical anthropology—and the diversity of the team contributed to maintaining balance in analysis and interpretation of data throughout the project.

### Ethical approval

This study was conducted with the approval of the Seoul National University Bundang Hospital Institutional Review Board (IRB; survey: IRB No. B-1808-484-308, semi-structured interview: IRB No. B-1809-492-303). All participants were given explanations regarding research ethics and signed informed consent after full knowledge on the aim of the study, benefit of participation, and withdrawal of participation. Identifiable personal information was deliberately deleted during the transcription process, and all participants were recorded on questionnaires and interview transcripts only as ID numbers.

## Survey results

### General characteristics, needs, state anxiety, and uncertainty in illness

The mean age of family members was 51.32 years, and the mean age of patients was 68.68. Mean ICU LOS was 12.96 days, and mean APACHE II score at the time of ICU admission was 21.96 (Table 1).

**Table 1:** General characteristics of family members and patients (*N* = 50).

			Mean±SD or N (%)
	Family’s characteristics		
Age (years)			51.32±11.28
	< 65		44 (88.0)
	≥ 65		6 (12.0)
Sex	Female		27 (52.9)
	Male		23 (45.1)
Relationship	Spouse		12 (23.5)
	Other		38 (74.5)
		Parents	4 (7.8)
		Sibling	3 (5.9)
		Offspring	30 (58.8)
		Relative	1 (2.0)
Living with	Yes		26 (51.0)
	No		24 (47.1)
	Patients’ characteristics		
Age			68.68±16.86
Sex	Female		13 (25.5)
	Male		37 (72.5)
LOS			12.96±13.40
Cause of admission	Sepsis/Septic shock		5 (10.0%)
	Respiratory Failure		38 (76.0%)
	Heart failure		1 (2.0%)
	Renal failure		3 (6.0%)
	Malignancy		1 (2.0%)
	Others		2 (4.0%)
Route of ICU admission	ER		14 (28%)
	Ward stay before ICU admission		36 (72%)
APACHE II score			21.96±7.39

SD = standard deviation; LOS = length of stay; ER = emergency room; ICU = intensive care unit; APACHE II = Acute physiology, age, chronic health evaluation II

The mean score for critical care family needs was 3.26 on a four-point scale. Among subscales, assurance was ranked at the top, followed by information, proximity, comfort, and support needs. The mean state anxiety score for family members was 52.84, and mean of uncertainty in illness score was 39.82. Further, 43.1% of participants showed symptoms of state anxiety (n = 22; Table 2).

**Table 2:** Family needs, state anxiety, and uncertainty of family members (*N* = 50).

		Mean±SD or N(%)	Range
Family needs	Total	3.26±0.40	2.27–3.98
	Assurance need	3.86±0.20	3.14–4.00
	Information need	3.38±0.37	2.33–4.00
	Proximity need	3.28±0.49	2.00–4.00
	Comfort need	2.96±0.63	1.50–4.00
	Support need	2.96±0.55	1.50–4.00
State anxiety		52.84±14.60	22.00–78.00
	≤ 51	22 (43.1)	
	≥ 52	22 (43.1)	
Uncertainty in illness		39.82±10.53	20.00–61.00

SD = standard deviation

### Critical care family needs, state anxiety, and uncertainty in illness according to general characteristics

Kolmogorov-Smirnov Z tests revealed that all variables of 0.611 (*p* = .849), 0.932 (*p* = .350), 1.221 (*p* = .101), 0.973 (*p* = .301), 0.679 (*p* = .745), 0.858 (*p* = .453), and 0.696 (*p* = .718), except assurance needs of 1.940 (*p* = .001), satisfied normality assumptions, and the Mann-Whitney U test, a nonparametric test, was conducted on assurance needs accordingly.

Results showed that there were no significant differences in assurance needs, with regard to general characteristics. For information needs, higher p-values were obtained when the family member was female (*p* = .040) or a spouse (*p* = .024). For proximity needs and comfort needs, higher p-values were obtained for females (*p* = .002, *p* = .029, respectively). For support needs, higher p-values were derived when the family member was female (*p* = .010) or a spouse (*p* = .019). For state anxiety and uncertainty in illness, no significant differences were found in general characteristics (Table 3).

**Table 3:** Family needs, state anxiety, and uncertainty according to general characteristics (*N* = 50).

	Age			Sex			Relationship			Living with		
	< 65	≥ 65	T or U(*p*)	F	M	T or U(*p*)	Spouse	Other	T or U(*p*)	Yes	No	T or U(*p*)
	Mean±SD	Mean±SD		Mean±SD	Mean±SD		Mean±SD	Mean±SD		Mean±SD	Mean±SD	
Assurance	3.87±0.19	3.83±0.29	118.50 (.820)	3.92±0.14	3.80±0.25	207.50 (.076)	3.94±0.10	3.84±0.22	162.50 (.164)	3.90±0.17	3.83±0.23	233.50 (.224)
Information	3.37±0.39	3.44±0.28	-0.478 (.635)	3.48±0.31	3.26±0.41	2.114 (.040)	3.58±0.24	3.30±0.39	2.337 (.024)	3.43±0.36	3.32±0.39	0.994 (.325)
Proximity	3.28±0.50	3.31±0.46	-0.164 (.870)	3.49±0.26	3.05±0.58	3.327 (.002)	3.43±0.27	3.24±0.54	1.617 (.115)	3.32±0.46	3.25±0.53	0.470 (.641)
Comfort	2.95±0.63	3.03±0.72	-0.266 (.791)	3.15±0.51	2.75±0.70	2.257 (.029)	3.18±0.58	2.89±0.64	1.382 (.174)	2.98±0.69	2.94±0.59	0.211 (.834)
Support	2.95±0.57	3.11±0.38	-0.643 (.523)	3.16±0.47	2.75±0.70	2.704 (.010)	3.32±0.45	2.87±0.54	.449 (.019)	3.05±0.52	2.88±0.59	1.064 (.293)
State anxiety	52.63±14.16	54.17±18.61	-0.237 (.814)	55.36±13.28	49.53±15.92	1.324 (.193)	59.25±11.86	50.44±14.96	1.832 (.074)	55.50±15.32	49.65±13.36	1.336 (.189)
Uncertainty in illness	39.72±9.74	40.50±16.33	-0.168 (.867)	42.50±9.04	36.78±11.43	1.952 (.057)	43.18±12.28	38.84±9.93	1.210 (.232)	40.44±12.16	39.17±8.72	0.420 (.677)

SD = standard deviation

### Correlations with state anxiety, uncertainty in illness, ICU LOS, APACHE II scores, and needs

A positive correlation between proximity needs and state anxiety (*p* = .027) was observed, whereas a significant negative correlation between comfort needs and uncertainty was found (*p* = .033). For state anxiety, significant positive correlations with uncertainty in illness and with LOS (*p* = .001 and *p* = .002, respectively) were observed. Similarly, uncertainty had a significant positive correlation with ICU LOS (*p* = .008; Table 4).

**Table 4:** Correlations with state anxiety, uncertainty, length of stay in ICU, APACH II scores, and family needs (*N* = 50).

		State anxiety	Uncertainty in illness	LOS	APACH II score
		*r* (*p*)	*r* (*p*)	*r* (*p*)	*r* (*p*)
Family needs	Total	.185 (.274)	.004 (.978)	.223 (.155)	.125 (.429)
	Assurance	.125 (.430)	.083 (.580)	.180 (.226)	.229 (.117)
	Information	.095 (.551)	-.074 (.623)	.222 (.138)	.192 (.197)
	Proximity	.346 (.027)	.077 (.607)	.288 (.050)	.171 (.252)
	Comfort	-.088 (.574)	-.308 (.033)	-.026 (.858)	.049 (.737)
	Support	.123 (.438)	.014 (.925)	.207 (.158)	.145 (.324)
State anxiety		1	.496 (.001)	.466 (.002)	.170 (.270)
Uncertainty in illness		.496 (.001)	1	.374 (.008)	.281 (.051)
LOS in ICU		.466 (.002)	.374 (.008)	1	-.001 (.992)
APACH II score		.170 (.270)	.281 (.051)	-.001 (.992)	1

LOS = length of stay; APACHE II = Acute physiology, age, chronic health evaluation II

### Interview findings

#### General characteristics of participants

The mean age of family members was 56.1 years, and the mean age of patients was 66.7. Mean ICU LOS was 11.3 days, and mean APACHE II score at the time of ICU admission was 24.3. Among 10 participants, 9 participants had religions; 4 participants were spouses, 4 participants were adult children; Whereas 7 patients were male, 7 participants were female. In terms of education level, 4 participants had bachelor’s or above degrees, and 6 participants had high school diploma (Table 5).

**Table 5:** Characteristics of study participants (Families) and critically Ill patients for depth semi-structured interviews (N = 10).

ID	Families					Patients				
	Gender	Age	Relationship	Education level	Religion	Gender	Age	Reason for admission ICU	ICU LOS (days)	Initial APACHE II Score
01	F	63	Spouse	High school diploma	Yes	M	63	Respiratory failure	33	16
02	F	44	Offspring	≥ Bachelor’s Degree	Yes	F	78	Renal failure	25	42
03	M	48	Offspring	≥ Bachelor’s degree	Yes	M	80	Respiratory failure	11	21
04	F	60	Sibling	High school diploma	Yes	F	62	Other	4	17
05	F	74	Spouse	High school diploma	Yes	M	76	Respiratory failure	2	26
06	F	51	Mother	High school diploma	Yes	M	20	Respiratory failure	3	31
07	F	67	Spouse	≥ Bachelor’s degree	No	M	71	Respiratory failure	6	19
08	M	41	Offspring	High school diploma	Yes	M	68	Respiratory failure	9	17
09	M	53	Offspring	≥ Bachelor’s degree	Yes	F	88	Respiratory failure	16	33
10	F	60	Spouse	High school diploma	Yes	M	61	Sepsis/Renal failure	4	21

ICU = intensive care unit; LOS = length of stay; APACHE = Acute physiology, age, chronic health evaluation

#### Response to ICU admission, anxiety, and uncertainty in illness

During in-depth interviews, state anxiety of family members of critically ill patients first presented as psychological shock about an ICU admission, viewing it as an unrealistic and unexpected event to happen in one’s own family. Interviewees associated ICU admission with the possibility of the patient’s death, and their anxiety intensified, especially when they were directly informed by medical staff of a negative prognosis or when they anticipated unfavorable outcomes of treatment based on their own observations. Further, concerns about the patient’s deteriorated quality of life after discharge (e.g., dialysis or severe disability) led to anxiety (No. 6, No. 7).

However, the ICU admission itself did not necessarily cause anxiety. Most interviewees expressed expectations and hopes for favorable outcomes, as they perceived the ICU as a place where patients could benefit from “machines” and “where the patient can receive dedicated intensive care” (i.e., a place in which medical knowledge and technology, as well as human resources, were concentrated) (No. 1, No. 3, No. 4, No. 7).

#### Information needs

All interviewees reported they communicated primarily with nurses and resident physicians in the ICU, mainly about the patient’s condition and future treatment plans. Regarding the understandability of terminology used by medical professionals, interviewees assessed that “it was not too hard to understand.” Specifically, one interviewee expressed satisfaction with supplementary images the attending nurse showed to help family members better understand how the treatment worked (No. 3). However, elderly interviewees reported they could not fully understand some explanations made by medical staff (No. 9, No. 10).

Interviewees expressed satisfaction with information that helped them comprehend the overall course and mechanisms of treatment, rather than fragmented information. They wanted to receive information that could meet their assurance needs or contribute to their feelings of control over uncertainties about the future as much as possible.

Not all interviewees wanted to receive all possible information. Some did not want to hear about unfavorable treatment outcomes (No. 1, No. 10), especially in front of the patients, because they thought an unfavorable prognosis might discourage patients and affect their recovery. Most interviewees reported they were able to ask medical staff any questions; however, some hoped the staff would explain them before the family members asked, because they did not want to interrupt the medical staff’s work (No. 1, No. 6). Most interviewees had a high need for direct communication with attending physicians, and some felt anxious about whether medical staff from diverse departments could collaborate and communicate with each other smoothly.

It should be noted that participants were not always sure whether the person with whom they had frequent communication was a nurse, resident, or attending physician. Information needs also emerged regarding joint decision-making with other family members. When discussing treatment plans and patient prognosis with family members, interviewees served as mediators who delivered information from the medical staff to other family members. Despite their efforts to “remember properly and convey accurately” what they heard from physicians and nurses, participants stated this task was not easy.

#### Support needs

Interviewees reported they received emotional support from other relatives and friends. Two interviewees were under extreme stress, due to their unmet needs for emotional support. One interviewee (son, No. 8) stated he had been the primary caregiver for a patient (mother) over the past 17 years, without having other family members to share the burden of care and decision-making. He was suffering from panic disorder. Another interviewee (wife, No. 2) was supposed to make decisions regarding her husband’s treatment with his seven siblings, under circumstances in which she financially relied on them. However, the patient’s siblings frequently reversed her decisions, coaxed her to relinquish her control over her husband’s medical treatment, and discouraged her by saying “he [patient] will die anyway.”

Regarding interactions with medical staff, one interviewee was stressed because she felt medical staff “forced” her to sign a consent form about withholding life-sustaining treatment (No. 1). Some interviewees responded that some programs offered by the hospital, such as cancer care counseling (No. 9) and social welfare programs (No. 2, No. 8), supported them.

Notably, financial support needs—which had not been included in the survey—emerged as an important theme in the interviews. With the exception of three interviewees, most participants complained of financial burdens caused by medical expenses and work loss, and exhibited high levels of satisfaction with the financial assistance program provided by the social welfare team.

#### Comfort needs

All interviewees (except No. 9) expressed dissatisfaction regarding comfort needs. Interviewees presupposed that they were willing to endure any inconveniences, if only patients could recover, and that they were grateful for a space arranged for the patient’s family. Nevertheless, several complaints were reported in relation to comfort needs, such as poor lounge conditions and inflexible ICU visiting hours that forced them to reorganize their personal schedule and travel long distances twice a day. It should be noted that comfort needs were ranked as a low priority compared to other needs on the survey, but were addressed in the interview with detailed comments.

#### Proximity needs

Some interviewees expressed feelings of guilt and helplessness, as there was nothing they could do for patients in severe pain (No. 4, No. 10). One interviewee (son, No. 8), who believed himself to be familiar with details of the patient (mother)’s history through long-time caregiving, wanted the medical staff to allow him to stay with the patient when her delirium worsened.

#### Assurance needs

Interviews made few direct statements regarding assurance needs, regardless of their strong wishes for the patients’ recovery. Rather than seeking assurance from the medical staff, interviewees managed to assure themselves by combining their own observation on the patient’s progress and information from the medical staff (No. 4).

#### Contribution of family-centered multidisciplinary rounds: Extending beyond information provision to encompass emotional support

Family-centered multidisciplinary rounds received positive evaluations from most participants, who exhibited high degrees of satisfaction, especially in terms of information needs. Particularly, the presence of multiple professionals was cited as strength of rounds, as this opportunity allowed them “to be informed of and ask questions about all relevant issues at once.” Interviewees reported family-centered multidisciplinary rounds provided an opportunity to communicate with the attending physician, which had been addressed as an unmet information need. Participants reported that family-centered multidisciplinary rounds alleviated their anxiety about whether medical staff from multiple departments could communicate smoothly with one another.

Family-centered multidisciplinary rounds were assessed as helpful for the family in making plans for future patient treatment and prognosis, as the multidisciplinary rounds were designed to bring together the ICU attending physician, resident, nurse, pharmacist, and social worker to discuss the patient’s clinical condition, current treatment status, and care plan. Participants also remarked that they could comprehend what the medical staff were discussing, because few medical and technical terms were used during rounds.

In addition to primary information that could be obtained from medical staff during family-centered multidisciplinary rounds, participants also highly appreciated meta-linguistic data obtained through the multidisciplinary format. The interviewees expected the rounds would be of great help not only to the patients and their families but also to attending physicians, in that the rounds would allow them to integrate more detailed and comprehensive information.

Properties of multidisciplinary rounds contributed to meeting information needs, and further providing emotional support. Some interviewees reported that had feelings of “trust” and “hope” when they witnessed medical staff from multiple professions discussing a patient.

## Discussion

This study was conducted to develop a Korean nursing model of family-centered critical care that satisfies needs of critical care family members. Previous studies conducted in South Korea have shown inconsistent results. There are some cases in which assurance needs are highest [27, 28], and other cases where proximity needs are highest [5]. Additionally, most studies regarding family members of critically ill patients in Korea have dealt with the correlation between stress and anxiety [29], or between strain and stress [5]; however, few have investigated the correlation between needs of family caregivers, anxiety, and uncertainty in illness. This study identified variables related to critical care family needs, state anxiety, and uncertainty in illness, in the context of family experience, via quantitative and qualitative methods.

Results showed that the mean of critical care family needs was lower than that of previous studies conducted in South Korea (3.26 vs. 3.4~3.5) [27, 28]. The means of uncertainty in illness and state anxiety were slightly lower than in previous studies [20, 30]. However, given that the state anxiety inventory, in practice, considers scores of 40 or above as clinical anxiety [30], the participants in this study could be regarded psychologically anxious. The semi-structured interview also indicated that family members of critically ill patients perceived ICU admission itself as a traumatic event and associated it with the possibility of death, which is consistent with findings of previous studies [31, 32]. The results of the present study showed that both anxiety and uncertainty in illness have positive correlations with ICU LOS, which suggested the need for psychological counseling for the families of long-stay patients.

Regarding subscale scores, assurance needs were ranked highest, followed sequentially by information needs, proximity needs, and comfort needs; this finding was consistent with previous studies [27]. Assurance needs obtained the highest scores regardless of general characteristics of patients or their families. Information needs were the second highest, as was suggested in a previous study [33] which found the need for information was universal and generic, independent of a family’s educational level or cultural background.

With regard to information needs, data from semi-structured interviews addressed some details of the contents and the ways of information delivery: first, participants exhibited satisfaction with information provided through supplementary materials. Second, participants did not seek merely fragmented information but more integrated information that covered both progress and prognoses of patients, so they could anticipate what would happen. Third, some participants were not satisfied with the discussion with medical staff regarding bad prognosis. Fourth, families of critically ill patients had some difficulties asking the working medical staff to provide information. These results underscore that, on the one hand, medical practitioners’ effort to engage family caregivers in clinical conversation is required, the quality of information and the way of conveying information impact the families’ information needs, on the other hand. As noted, communication strategy in delivering bad news and discussing advance care planning should be tailored according to the patient and the family members’ socio-cultural backgrounds [6; 34, 35]. Our study also highlights the necessity of sensitive approach to the relatives especially in consideration of age and expectation of a patient’s recovery.

Semi-structured interviews revealed that joint decision-making could incur additional burdens to the primary caregiver: (1) mediation between family members and (2) information conveyance from the medical staff to other family members. We suggest the use of ICU diaries as a resolution of the information conveyance issue. The difficulty of mediation was addressed particularly in cases where the primary caregiver was not financial stable. A preliminary interview with the social worker who participated in the family-centered multidisciplinary rounds offered an opportunity to identify the family conflict and economic hardships. Medical staff may consider empowering the primary caregiver during multidisciplinary rounds by elaborating caring atmosphere and discussing attitude and behavior [36], if he or she seems highly dependent on other joint-decision makers.

In this study, there were differences in need of family according to gender. Female family members of critically ill patients had higher information, proximity, comfort, and support needs, compared to male family members. These results consistent with previous study [37] that reported female caregivers may feel more obligated and expected to fulfill a wide variety of caregiving roles.

In terms of the effect of visiting hours, previous studies have found incongruent results: some studies have reported that extended patient visiting hours lowered levels of depression and anxiety [38], while others have reported that the same measure did not contribute meeting critical care families’ proximity needs [39]. These conflicting findings indicated that proximity needs do not simply mean a desire to be physically close to a patient. As revealed in interviews, participants wanted to contribute to patients’ care, even if the action was very trivial. Thus, it is necessary to consider a wide variety of visiting methods as well as more flexible visiting hours, beyond passive visits and extension of ICU visiting hours. Given that various key factors affect the involvement of critical care family members in nursing care, including family characteristics, workload of nurses, severity of the patient’s illness, safety for patients and their families, and legal issues and that nurses tend to be unaware of how to engage family members in critical care nursing [40], this research data emphasize the necessity of a paradigm shift to study the scope and types of proximity needs, the efficacy of families’ active involvement in alleviating their helplessness, and standard protocols. In Korea, meeting with family caregivers are mainly conducted by doctors and nurses, but it is also needed for various medical staffs such as physiotherapists to participate in the meeting with family caregivers and educate them about the procedures that caregivers could do.

In this study, comfort needs showed a negative correlation with uncertainty in illness. It should be noted that comfort needs were ranked as a low priority compared to other needs in the survey, but were addressed in the interviews with specific comments on visiting hours and poor family lounge conditions. This indicates that comfort needs, if not a high priority, are real issues for critical care families. As some studies have reported that comfort needs were linked to ICU settings, and satisfying the comfort needs consequentially led to family satisfaction [41], the lounge area for critical care families may also deserve attention.

All participants (except No. 1) exhibited positive responses to family-centered multidisciplinary rounds, which was consistent with findings from previous studies [42–44]. Family-centered multidisciplinary rounds enabled them to obtain high-quality information directly from multiple professionals that contained both what treatment the patient had received and would receive, and this comprehensive information helped participants to predict the future events despite uncertainty in illness. Furthermore, participants were not merely passive recipients of information, but evaluated meta-information in a way that such a format of rounds would facilitate the medical staff, especially attending physicians, to establish and implement patient-tailored care plans. Therefore, future family-centered multidisciplinary rounds should focus on sharing treatment plans with, and offering question and answer opportunities to, family caregivers.

Many participants had trouble telling with whom they had communicated, which implied a lack of proper introductions in Korean ICU settings. In comparison, they had a clear idea of who participated in family-centered multidisciplinary rounds, because the medical staff introduced themselves by position at the beginning. Thus, we expect that family-centered rounds can contribute to enhancing the quality of communication between medical staff and families. However, it is also of note that the communicative factors adopted by family-centered rounds, especially discussing the bad prognosis in front of the patient, may be not familiar to some family members. This reluctance to discuss unfavorable prognoses with patients is dominant not only in Korea but also in many countries [45]. In order to save the advantage from the family-centered rounds, it is necessary to fully explain, in advance, the benefits of involving both patients and their families in treatment plan discussions and to obtain consent accordingly. Also, the social work counseling services included in the multidisciplinary rounds protocol provided not only financial assistance but also emotional support for critical care families, which could be particularly essential to family members of critically ill patients who lack a support system.

Some limitations of the present study should also be noted. A major limitation of this study is that it included only family members of critically ill patients admitted to the medical ICU. It may be that family members in other ICU settings have different experiences and needs. Moreover, since the family-centered multidisciplinary rounds took place in the morning, this study could not recruit family members who had to go to work during the day.

## Conclusion

In the present study, female family members of critically ill patients had higher information, proximity, comfort, and support needs, compared to male family members, and spouses had higher information and support needs, compared to other family members. Proximity needs were positively correlated with state anxiety, and comfort needs were negatively correlated with uncertainty in illness. The families of critically ill patients who participated in family-centered multidisciplinary rounds stated positively that this allowed them to understand overall treatment plans and facilitated communication with medical staff. Based on these findings, a family-centered multidisciplinary rounds protocol can be established and implemented and, as the next step, further intervention studies are recommended to conduct a forward-looking assessment regarding the effects of family-centered multidisciplinary rounds on critical care family needs.

## Supporting information

S1 Data (XLSX)

S1 File (DOCX)

S1 Questionnaire (DOCX)
