# Supplementary material for: State anxiety, uncertainty in illness, and needs of family members of critically ill patients and their experiences with family-centered multidisciplinary rounds: A mixed model study
Source: PLoS One. 2020 Jun 9;15(6):e0234296. doi: 10.1371/journal.pone.0234296 (PMC7282650; doi:10.1371/journal.pone.0234296)
Supplement: S1 File — (DOCX) [file pone.0234296.s002.docx]

**Interview Guide (English Version)**

1. Psychological regarding admission of intensive care units and care for patients

- How did the patient get into intensive care? Could you describe the day?

- What did you think when you heard of the patient’s admission to intensive care? What was your biggest worry?

- Who do participate in the care work for the patient?

2. Physical and economic burdens

- How long have you been caring for the patient?

- How do you (a family caregiver) take a rest?

- What is the biggest obstacle to care for the patient?

- Who does pay for the hospital bill?

3. Establishing a family care plan

- Who are sharing the care labor?

- How does each family member feel about care for the patient?

4. Communications with the medical staff

- What kind of topic did you usually talk with the nurse/resident/attending doctor? Were you able to convey what you wanted to address to the medical staff? Did you understand the nurse/resident/attending doctor?

5. Evaluation of care

- Do you have any suggestions to the hospital and the medical staff in the ICU?

- What do you feel most sorry for during the patient’s hospitalization in the ICU?

**Interview Guide (Korean Version)**

1. 중환자실 입실과 돌봄 과정에서 겪은 심리적 충격 및 극복

- 어떻게 환자가 중환자실로 입실하게 되었습니까?

- 처음 중환자실 입실에 대해 설명 들을 때 어떤 생각이 들었습니까? 무엇이 가장 큰 걱정이었나요?

- 누가 환자의 돌봄에 참여하고 있습니까?

2. 중환자실에 재원한 환자를 돌보는 것에 대한 신체적, 경제적 부담 및 극복

- 얼마 동안 환자를 돌보고 있습니까?

- 돌봄제공자가 휴식을 취하는 시간과 방법은 무엇입니까?

- 어떤 점이 환자 돌봄에 가장 큰 걸림돌입니까?

- 병원비는 누가 부담하고 있습니까?

3. 가족 내 돌봄 계획 수립

- 누가 교대로 환자를 돌보고 있었습니까?

- 각 구성원들은 환자 돌봄에 대해 어떤 태도를 가지고 있습니까?

4. 중환자실 의료진과의 커뮤니케이션 장애

- 담당 간호사/레지던트/의사와 어떤 이야기를 주로 했습니까? 하고 싶은 이야기를 충분히 전달했습니까? 의료진의 이야기는 이해할 수 있었습니까?

5. 중환자실에서 받은 돌봄에 대한 평가

- 병원 측에 건의하고 싶은 사항이 있습니까?

- 중환자실에 환자가 있는 동안 무엇이 가장 아쉽고 속상합니까?
