# Supplementary material for: State anxiety, uncertainty in illness, and needs of family members of critically ill patients and their experiences with family-centered multidisciplinary rounds: A mixed model study
Source: PLoS One. 2020 Jun 9;15(6):e0234296. doi: 10.1371/journal.pone.0234296 (PMC7282650; doi:10.1371/journal.pone.0234296)
Supplement: S1 Questionnaire — (DOCX) [file pone.0234296.s003.docx]

1. **귀하에 대한 정보**

| 1. **나이(만)** |  |
| --- | --- |
| 1. **성별** | **□ 남 □ 여** |

1. **환자 정보**

| 1. **나이(만)** |  |
| --- | --- |
| 1. **성별** | **□ 남 □ 여** |
| 1. **중환자실 면회 횟수** | **□1회 □2회 □3회 □4회이상** |

1. **귀하에 관한 정보**

| **환자와의 관계** | **□** 배우자 **□** 동거인 **□** 부모 **□** 형제, 자매  **□** 자녀 **□** 친척 **□** 친구 **□** 기타: ________ |
| --- | --- |
| **환자와 동거 유무** | **□** 예 **□** 아니오 |
| **종교** | **□** 가톨릭 **□** 기독교 **□** 불교  **□** 무교 **□** 기타: __________ |
| **월평균 수입** | **□** 100만원 미만 **□** 100-200만원 **□** 200-300만원 **□** 300-400만원  **□** 400-500만원 **□** 500만원 이상 |
| **주 수입원** | **□** 환자 **□** 환자 외 다른 가족(환자와의 관계): ______ ( ) |
| **교육 정도** | **□** 무학 **□** 초,중졸 **□** 고졸 **□** 대졸 이상 |
| **직업** | **□** 회사원/사무직 **□** 기업인/경영직 **□** 판매/서비스직  **□** 공무원 **□** 교사/교수 **□** 자유직/프리랜서  **□** 자영업 **□** 전문직 **□** 생산(농,어업)  **□** 학생 **□** 주부 **□** 기능/노무  **□** 기타: ________ |
| **환자 이외에 다른 가족의 중환자실 입원 여부** | **□** 예 **□** 아니오  “예”라고 답하셨을 경우 그 가족 구성원의 중환자실 입원 횟수: ( )회 |

1. **다음 문항은 귀하의 가족이 중환자실에 입원하고 있는 동안 보호자로서 느끼는 요구에 대한 내용입니다. 각 문항의 중요도를 오른쪽 칸에 √ 표를 해 주시기 바랍니다.**

| No | 내용 | 매우 중요하다 | 중요하다 | 약간중요하다 | 중요하지 않다 |
| --- | --- | --- | --- | --- | --- |
| 1 | 환자에게 예상되는 결과를 아는 것은 중요합니까? |  |  |  |  |
| 2 | 처음 중환자실에 입원하기 전 중환자실 환경에 대해 설명을 듣는 것은 중요합니까? |  |  |  |  |
| 3 | 매일 담당의사와 면담하는 것은 중요합니까? |  |  |  |  |
| 4 | 보호자가 면회 올 수 없을 때 연락할 수 있는 중환자실 담당자의 연락처를 아는 것은 중요합니까? |  |  |  |  |
| 5 | 질문에 대해 의료진이 솔직한 답변을 해 주는 것은 중요합니까? |  |  |  |  |
| 6 | 특수한 환자 상태의 경우 정규면회시간 이외에 면회가 가능한 것은 중요합니까? |  |  |  |  |
| 7 | 환자에게 일어난 일에 대해 나의 감정을 이야기하는 것은 중요합니까? |  |  |  |  |
| 8 | 병원 내에 보호자를 위한 좋은 식음료 시설이 있는 것은 중요합니까? |  |  |  |  |
| 9 | 보호자가 침상 옆에서 환자에게 해줄 수 있는 일을 안내 받는 것은 중요합니까? |  |  |  |  |
| 10 | 보호자의 필요에 따라 면회시간을 조정할 수 있는 것은 중요합니까? |  |  |  |  |
| 11 | 각 의료진이 어떤 정보를 제공할 수 있는지 아는 것은 중요합니까? |  |  |  |  |
| 12 | 보호자를 지지해 줄 수 있는 지인이 주변에 있는 것은 중요합니까? |  |  |  |  |
| 13 | 환자에게 제공되는 처치나 치료가 왜 이루어졌는지 아는 것은 중요합니까? |  |  |  |  |
| 14 | 희망이 있다고 느끼는 것은 중요합니까? |  |  |  |  |
| 15 | 환자를 돌보는 다양한 직종의 의료진 (의사, 간호사, 영양사, 물리치료사 등)에 대해 아는 것은 중요합니까? |  |  |  |  |

| No | 내용 | 매우 중요하다 | 중요하다 | 약간중요하다 | 중요하지 않다 |
| --- | --- | --- | --- | --- | --- |
| 16 | 환자가 어떤 치료를 받는지 아는 것은 중요합니까? |  |  |  |  |
| 17 | 환자에게 가능한 최상의 치료가 제공되고 있다는 확신을 받는 것은 중요합니까? |  |  |  |  |
| 18 | 병원 내에 보호자를 위한 독립된 공간이 있는 것은 중요합니까? |  |  |  |  |
| 19 | 환자를 위해 어떤 일들이 이루어지고 있는지 정확히 아는 것은 중요합니까? |  |  |  |  |
| 20 | 보호자 대기공간에 편안한 가구가 마련되어 있는 것은 중요합니까? |  |  |  |  |
| 21 | 병원 직원들이 가족에게 우호적인 것은 중요합니까? |  |  |  |  |
| 22 | 의료비와 관련된 문제를 도와줄 수 있는 사람이 있는 것은 중요합니까? |  |  |  |  |
| 23 | 보호자 대기공간에 무료 와이파이 (무선 인터넷), 핸드폰 충전 서비스 등이 제공되는 것은 중요합니까? |  |  |  |  |
| 24 | 목사님, 신부님, 스님 등의 종교지도자가 방문하는 것은 중요합니까? |  |  |  |  |
| 25 | 의료진과 환자의 사망 가능성에 대해 이야기 할 수 있는 것은 중요합니까? |  |  |  |  |
| 26 | 중환자실을 방문할 때 누군가와 함께 동반 면회가 가능한 것은 중요합니까? |  |  |  |  |
| 27 | 보호자의 건강에 대해 염려하는 사람이 있는 것은 중요합니까? |  |  |  |  |
| 28 | 보호자가 잠시 병원에 없어도 괜찮다는 확신을 받는 것은 중요합니까? |  |  |  |  |
| 29 | 매일 같은 담당 간호사와 대화 할 수 있는 것은 중요합니까? |  |  |  |  |
| 30 | 울어도 괜찮다고 느끼는 것은 중요합니까? |  |  |  |  |
| 31 | 어려움이 있을 때 도와줄 수 있는 담당자에 대한 정보를 제공 받는 것은 중요합니까? |  |  |  |  |
| 32 | 보호자 대기공간 가까이에 화장실이 있는 것은 중요합니까? |  |  |  |  |
| 33 | 원할 때 혼자 있을 수 있는 것은 중요합니까? |  |  |  |  |
| 34 | 가족 문제가 있을 때 도와줄 수 있는 담당자에 대한 정보를 제공 받은 것은 중요합니까? |  |  |  |  |
| No | 내용 | 매우 중요하다 | 중요하다 | 약간중요하다 | 중요하지 않다 |
| 35 | 이해하기 쉽게 설명해 주는 것은 중요합니까? |  |  |  |  |
| 36 | 면회가 정해진 시간에 시작하는 것은 중요합니까? |  |  |  |  |
| 37 | 종교 서비스에 대한 정보를 제공 받는 것은 중요합니까? |  |  |  |  |
| 38 | 환자를 돌보는 것 (세수, 머리감기, 양치 등)을 도울 수 있게 해주는 것은 중요합니까? |  |  |  |  |
| 39 | 병실 이동 계획이 있는 경우, 정보를 제공받는 것은 중요합니까? |  |  |  |  |
| 40 | 정규면회시간 이외에 환자의 상태 변화에 대한 정보를 전화로 제공받는 것은 중요합니까? |  |  |  |  |
| 41 | 적어도 하루에 한 번은 환자 상태에 대한 정보를 제공받는 것은 중요합니까? |  |  |  |  |
| 42 | 병원직원들이 환자에게 관심이 있다고 느끼는 것은 중요합니까? |  |  |  |  |
| 43 | 환자의 경과에 대해 구체적인 사실을 아는 것은 중요합니까? |  |  |  |  |
| 44 | 환자를 자주 볼 수 있는 것은 중요합니까? |  |  |  |  |
| 45 | 보호자 대기공간이 중환자실 가까이에 있는 것은 중요합니까? |  |  |  |  |

46. 상기 문항 외에 중환자 가족으로서 중요하다고 생각하는 사항을 자유롭게 적어 주십시오.

(

)

**5. 다음은 현재 느끼고 계시는 감정에 대한 질문입니다. 해당된다고 생각하시는 번호에 표시에 주십시오.** 상태불안 (Spielberger, 1970) (김정택, 신동균, 1978, 번역 표준화)

| 문항 | 전혀  그렇지  않다 | 조금  그렇다 | 보통으로 그렇다 | 대단히 그렇다 |
| --- | --- | --- | --- | --- |
| 1. 나는 마음이 차분하다. |  |  |  |  |
| 2. 나는 마음이 든든하다. |  |  |  |  |
| 3. 나는 긴장이 되어있다. |  |  |  |  |
| 4. 나는 후회스럽고 서운하다. |  |  |  |  |
| 5. 나는 당황해서 어찌할 바를 모르겠다. |  |  |  |  |
| 6. 나는 앞으로 불행이 있을까봐 걱정하고 있다. |  |  |  |  |
| 7. 나는 마음이 편하다. |  |  |  |  |
| 8. 나는 마음이 놓인다. |  |  |  |  |
| 9. 나는 불편하다. |  |  |  |  |
| 10. 나는 편안하게 느낀다. |  |  |  |  |
| 11. 나는 자신감이 있다. |  |  |  |  |
| 12. 나는 짜증스럽다. |  |  |  |  |
| 13. 나는 마음이 조마조마하다. |  |  |  |  |
| 14. 나는 극도로 긴장되어 있다. |  |  |  |  |
| 15. 내 마음은 긴장이 풀려 푸근하다. |  |  |  |  |
| 16. 나는 만족스럽다. |  |  |  |  |
| 17. 나는 걱정하고 있다. |  |  |  |  |
| 18. 나는 흥분되어 어쩔 줄 모르겠다. |  |  |  |  |
| 19. 나는 즐겁다. |  |  |  |  |
| 20. 나는 기분이 좋다. |  |  |  |  |

**6. 질병 불확실성 측정 도구 (Mishel, 1988 이 개발한 질병 불확실성 척도, Mishel Uncertainty in Illness Scale, MUIS, 조옥희, 2000 수정 보완. 보호자 용으로 수정)**

**다음 문장을 읽고 각 질문에 대한 자신의 느낌과 생각에 해당되는 곳에 0표를 하십시오.**

|  | 전혀 그렇지 않다 | 대체로 그렇지 않다 | 보통이다 | 대체로 그렇다 | 매우 그렇다 |
| --- | --- | --- | --- | --- | --- |
| 1. 환자 몸의 무엇이 나쁜지 잘 모르겠다. |  |  |  |  |  |
| 2. 나는 알고 싶은 질문이 많다. |  |  |  |  |  |
| 3. 환자 병이 나아가는 것인지 깊어 가는 것인지  잘 모르겠다. |  |  |  |  |  |
| 4. 환자가 겪을 불편감이 얼마나 심할지 모르겠다. |  |  |  |  |  |
| 5. 환자 상태에 대해 의료진이 설명하는 것이 애매모호하다. |  |  |  |  |  |
| 6. 내 기대가 언제 이루어질지 모르겠다. |  |  |  |  |  |
| 7. 의료진이 내게 설명한 내용을 모두 이해할 수 있다. |  |  |  |  |  |
| 8. 환자 병에 대한 치료방법이 매우 복잡하다고 생각한다. |  |  |  |  |  |
| 9. 지금 행해지고 있는 검사나 치료법이 환자에게 도움이 될지 모르겠다. |  |  |  |  |  |
| 10. 환자 병의 경과를 잘 모르므로 앞일을 계획할 수 없다. |  |  |  |  |  |
| 11. 치료가 모두 끝난 후 내가 어떻게 해야 할 지 모르겠다. |  |  |  |  |  |
| 12. 환자에게 어떤 일이 일어나고 있는지 잘 모르겠다. |  |  |  |  |  |
| 13. 의료진들이 환자 병의 치료계획에 대해 말하지 않았다. |  |  |  |  |  |
| 14. 환자가 스스로 돌볼 수 있을 때까지 얼마나 오래 걸릴지 모르겠다. |  |  |  |  |  |
| 15. 나는 환자 병의 경과를 대체로 예측할 수 있다. |  |  |  |  |  |
| 16. 나는 환자 체력이 결국은 회복되리라고 확신한다. |  |  |  |  |  |
